# Study on the correlation parameters of Class II malocclusion in child tooth dentition early stage by using 3D technique

**DOI:** 10.12669/pjms.35.5.592

**Published:** 2019

**Authors:** Peiying Xiong, Wen Sui, Wendan He, Qiaoling Lei, Wenli Gao

**Affiliations:** 1 Peiying Xiong, Department of Stomatology, Shenzhen Hospital of Southern Medical University, Shenzhen, 518000, China; 2 Wen Sui, Department of Stomatology, Shenzhen Hospital of Southern Medical University, Shenzhen, 518000, China; 3 Wendan He, Department of Stomatology, Shenzhen Hospital of Southern Medical University, Shenzhen, 518000, China; 4 Qiaoling Lei, Department of Stomatology, Shenzhen Hospital of Southern Medical University, Shenzhen, 518000, China; 5 Wenli Gao, Department of Stomatology, Shenzhen Hospital of Southern Medical University, Shenzhen, 518000, China

**Keywords:** 3D technique, Children, Dental transition, Class II malocclusion

## Abstract

**Objective:**

To record the dentition, jaw and facial growth and development of children with class II malocclusion at the age of 7-8 years old in the early dental transitional stage with 3D technology and to provide the study basis for the growth and development parameters of normal children and children with class II malocclusion.

**Methods:**

Twenty-four children who were suffering class-II malocclusion in the early dental transitional stage and received treatment between July 2016 and July 2017 in our hospital were selected as the study group, and 20 healthy children were selected as the control group in the same period. SIRONA CEREC dentition scanning, 3D reconstruction of the lower mandible and 3d MD face scanning were performed on the children. Relevant data were recorded and compared.

**Results:**

The dentition scanning results suggested that the study group had significantly larger anterior overbite and anterior overjet and smaller width of the upper arch than the control group; there was a significant difference between the two groups (P<0.05). The 3D reconstruction of the lower mandible suggested that the study group had smaller Go angle and SNB angle and shorter ANS-Me distance, Go-Me distance and N-Me distance compared to the control group; the differences had statistical significance (P<0.05). The face scanning results demonstrated that the nasolabial angle and facial convexity angle of the study group were significantly larger than those in the control group, and the difference was statistically significant (P<0.05).

**Conclusion:**

The dentition scanning results suggested that the study group had significantly larger anterior overbite and anterior overjet and smaller width of the upper arch than the control group; there was a significant difference between the two groups (P<0.05). The 3D reconstruction of the lower mandible suggested that the study group had smaller Go angle and SNB angle and shorter ANS-Me distance, Go-Me distance and N-Me distance compared to the control group; the differences had statistical significance (P<0.05). The face scanning results demonstrated that the nasolabial angle and facial convexity angle of the study group were significantly larger than those in the control group, and the difference was statistically significant (P<0.05).

## INTRODUCTION

Malocclusion is one of the three major oral epidemic diseases announced by the World Health Organization (WHO). Class II malocclusion which has an incidence of 5%~8% is a common malocclusion in clinics.^1^ Malocclusion is not a disease, but it not only can affect the appearance, but also is closely related to a series of oral problems.^2^ If timely intervened was not given, it will affect the development of the stomatognathic system and the health of temporal-mandibular joint.

In the growth and development stage of children, dentition, jaw and facial shape develops rapidly, and malocclusion is easy to occur in that stage.^3^ Many studies in China and abroad have shown that there are differences in tooth and craniofacial morphology and developmental characteristics between different races, regions and genders.^4^ Therefore, it is of great significance for local clinical diagnosis and treatment to obtain the dentition, facial pattern and mandible measurement values of local boys and girls in different areas. In the early occlusive induction treatment of dental transitional stage, it is necessary to take into account the characteristics of the growth and development of the teeth and arch of children and adolescents, and the design of clinical treatment should adapt to it. Therefore, twenty-four children with class II malocclusion in early dental transitional stage who received treatment between July 2016 and July 2017 were selected as the study group, and 20 healthy children in the same period were selected as the control group. The dentition, jaw and facial growth and development conditions and relevant parameters were recorded using 3D digital technology. This work provides a research basis for the growth and development parameters of normal children and children with class II malocclusion in the dental transitional stage.

## METHODS

Twenty-four children with class II malocclusion in the dental transitional stage who received treatment between July 2016 and July 2017 were selected as the study group. The inclusive criteria included erupted central incisor in the upper and lower jaws and erupted first molar in the bilateral upper and lower jaws, complete detition, abnormal upper and lower anterior teeth inclination, grade II~III anterior overjet, distal occlusion of bilateral first molar, lateral face protrusion or mandibular retrusion and uncoordinated facial height to width ratio. Familial inherited bone deformity, rampant caries, class III malocclusion, congenital or acquired teeth number and shape abnormality and growth and development diseases were excluded. Moreover twenty healthy children who aged seven or eight years were selected as the control group. They had complete dentition, normal upper and lower anterior teeth inclination, grade I anterior overjet, Grade I~II anterior overbite, neutroclusion of bilateral first molar, coordinated lateral face and coordinated facial height to width ratio. The study group included 15 boys and 9 girls, and they aged from 7~8 years (average (7.8±2.1) years). The control group included 13 boys and 7 girls, and they aged from 7~8 years (average (7.5±2.3) years). No significant difference was found in the general data between the two groups (P>0.05). The experimental program has been approved by the medical ethics committee of our hospital, and the guardians of all the children have fully understood the content of the program and agreed to join the experiment.

### Methods

Non-invasive dentition 3D scanning, 3D reconstruction of the lower mandible and 3D face scanning were carried out on children in the two groups, and the detection data were recorded. The width of the upper dental arch (width of mesiobuccal cusp of the first molar of bilateral maxilla) and the anterior overbite and overjet quantity were measured using SIRONA CEREC orthodontics analysis software. The morphological parameter of mandibular angle was measured using Sim Plant Pro11.04. The variation tendency of nasolabial angle and facial convexity angle were recorded using 3d MD face scanner. The dentition, the morphological parameters of the mandible and facial soft tissue parameters were compared and analyzed.

### Data Analysis

All the data were analyzed using SPSS 20.0. Categorical data were expressed as percentage (%). Measurement data were expressed by mean ± standard deviation. Mean difference was analyzed using independent sample t test. Difference was considered as statistically significant if P<0.05.

## RESULTS

The scores of the anterior overbite and overjet of the study group were significantly higher than those of the control group, and the differences were statistically significant (P<0.05). The upper dental width (width of mesiobuccal cusp of the first molar of bilateral maxilla of the control group was larger than that of the study group, and the difference had statistical significance (P<0.05, Table-I).

**Table I:** The comparison of 3D scanning data analysis results between the two groups.

Group	Study group	Control group	t	P
Anterior overbite	3.32±0.31	1.62±0.18	4.167	<0.05
Anterior overjet	3.45±0.36	2.12±0.26	5.238	<0.05
Width of the upper arch (mm)	47.53±2.52	48.73±2.87	3.149	<0.05

The Go angle and SNB angle of the study group were smaller than those of the control group. The ANS-ME distance, Go-Me distance and N-Me distance of the study group were shorter than those of the control group. The Pog-NB distance of the study group was longer than that of the control group, and the difference was statistically significant (P<0.05, Table-II).

**Table II:** Comparison of 3D mandible reconstruction data analysis results between the two groups.

Group	Study group	Control group	t	P
Go (°)	122.21±6.79	126.24±5.41	5.653	<0.05
SNB (°)	75.85±2.08	79.06±2.03	2.651	<0.05
ANS-Me (mm)	51.64±5.78	54.23±2.18	4.712	<0.05
Go-Me (mm)	73.65±5.67	77.13±3.89	3.332	<0.05
N-Me (mm)	112.36±6.48	115.15±5.26	2.568	<0.05
Pog-NB (mm)	2.14±1.67	1.20±1.26	6.617	<0.05

The study group had larger nasolabial angle and smaller facial convexity angle compared to the control group, and the differences had statistical significance (P<0.05, Table-III).

**Table III:** Comparison of facial 3D scanning data analysis results between the two groups.

Group	Study group	Control group	t	P
Nasolabial angle (°)	94.22±5.89	92.01±5.23	2.168	<0.05
Facial convexity angle (°)	132.25±5.84	130.31±4.83	2.057	<0.05

## DISCUSSION

Childhood is an important stage of oral development. Children’s living habits and other external factors can affect the development of teeth and jaws, leading to malocclusion.^5^,^6^ Although malocclusion does not directly result in children’s life safety, it can affect children’s facial image and has an important impact on children’s psychological development and life.^7^,^8^ According to the survey of Chinese Medical Association, the total prevalence of oral malocclusion was 67.82%, the incidence of oral malocclusion in the primary dentition stage was 51.82%, the incidence in the dental transitional period was 71.21%, and the incidence in the permanent dentition was 72.92%. It can be seen that the incidence of malocclusion increases from the deciduous teeth stage to the permanent dentition stage, indicating that the incidence of malocclusion increases with the growth and development of children. Moreover it also suggests that relevant parameters change constantly in the growth and development period, which aggravates the occurrence of oral malocclusion. The development period of children is a key period for treatment.^9^

At present, it is generally believed that the permanent dentition stage is the key period for the treatment of malocclusion, ignoring the attention to malocclusion in the dental transitional period.^10^,^11^ Research on the parameters of dentition and maxillofacial development, diagnostic criteria, diagnostic principles and treatment schemes for adolescents (11-13 years old) in the permanent dentition period have been mature.^12^,^13^ However, there are few reports on the growth and development parameters of dentition, jaw and soft tissue in the early dental transitional period (7-8 years old), and it is difficult for children to complete the orthodontic treatment process, resulting in poor orthodontic treatment outcome for most of the children.^14^

The children who miss the opportunity of early orthodontic treatment will have serious malocclusion and maxillofacial malformation, and some patients even need orthodontic surgery in adulthood, which not only increases the difficulty of treatment and treatment time, but also increases the treatment complications and treatment costs. In recent years, 3D technology has been more and more used in the medical field, and there are some studies about craniofacial plastic surgery.^15^,^16^ 3D technology can provide patients with personalized treatment programs and improve the visualization and accuracy of treatment.^17^ Shen simulated the oral surgery environment through digital 3D simulation, carried out surgical simulation,^18^ and achieved good results. Hu et al. found that direct digital manufacturing HD a promising future in oral and maxillofacial surgery.^19^

In this study, the growth and development of dentition, jaw and facial morphology of children with malocclusion and normal children were compared and analyzed through intraoral scanning, 3D reconstruction of mandible and 3D facial scanning. The results showed that the scores of anterior overbite and overjet in the study group were significantly higher than those in the control group and the upper arch width of the control group (the width of the mesiobuccal cusp of the bilateral maxillary first molars) was significantly larger than that of the study group; the differences were statistically significant. It indicated that children with malocclusion had crowded dentition and abnormal tooth morphology. In addition, malocclusion cannot only change oral parameters, but also change the development of the jaw, especially the mandibular morphology and facial parameters.^20^,^21^

The results of 3d MD face scanning showed that the nasolabial angle and facial convexity angle of the control group were smaller than those of the study group, and the differences were statistically significant. Compared with normal children, children with class II malocclusion had insufficient development in the lower 1/3 of the face, underdeveloped length of the mandible, underdeveloped alveolar point of the mandible, flatter mandibular angle, and more prominent mandibular chin, and all the differences were statistically significant, which was similar to the research results of El Hajj et al.^22^ It might be because that the lingual inclination and deep malocclusion of anterior teeth inhibited the growth and development of the mandible alveolar bone^23^ and led to a large difference of NB plane. The inference conformed to the relevant research results of a previous study.^24^ Tian et al. pointed out most cases of class II malocclusion was induced by retraction or shorter lower jawbone. Therefore the characteristics of class II malocclusion should be clearly understood in the early treatment. The key of the treatment is to open articulation to avoid the inhibitory effect of the upper anterior teeth on the development of the mandible.

### Limitations of the study

This study is single-center and small-sample, therefore it might ignore the influence brought by the difference of people from different regions. Hence the future study needs more multi-center and large-sample controlled trial to provide more reliable guidance basis for clinics.

## CONCLUSION

This work provides data support for the 3D diagnosis of malocclusion of children in the dental transitional period through detecting the dentition, jawbone and facial morphology parameters using 3D technology. The advantage of this study is that the 3D model measurement effectively reduced fixed point and measurement error, increasing the accuracy of the results, and moreover it proved that the monitoring and treatment of malocclusion should be paid attention to in the dental transitional period.

## References

[1] Zhang Y, Wang L, Zhao CY (2011). Influence of functional correction device on soft tissue profile of class II division I malocclusion. Stomatol.

[2] Zhang JX (2016). Influence of different degrees of malocclusion on the oral health related life quantity. Matern Child Health Care China.

[3] Giannattasio A, Poggi E, Migliorati M, Mondani PM, Piccardo I, Carta P (2015). The efficacy of Italian guidelines in promoting oral health in children and adolescents. Eur J Paedatr Dent.

[4] Bastir M, O'Higgins P, Rosas A (2007). Facial ontogeny in Neanderthals and modern humans. Proc Biol Sci.

[5] Zhang AX (2014). Observation and analysis of correlation between bad oral habits and malocclusion. Chin J Mod Drug Appl.

[6] Liu Y (2016). Exploration of the correlation between malocclusion of 6~10-year old children and bad oral habits. Matern Child Health Care China.

[7] Wang S (2013). Early prevention and treatment of childhood malocclusion. Chin J Pract Stomatol.

[8] Winter BU, Stenvik A, Vandevska-Radunovic V (2009). Dynamics of orthodontic root resorption and repair in human premolars:a light microscopy study. Eur J Orthod.

[9] Marques LS, Ramos-Jorge ML, Rey AC, Armond MC, Ruellas AC (2010). Severe root resorption in orthodontic patients treated with the edgewise method:prevalence and predictive factors. Am J Orthod Dentofac Orthop.

[10] Wang X, Wu CF, Li YS, Wang R (2014). Research progress on relationship between malocclusion as well as orthodontic treatment and psychosocial behavior. Chin Med Herald.

[11] Yang T, Li Y, Wang K, Wu XG, Yan XJ (2014). Analysis on the correlation between malocclusion and phonetic function. Matern Child Health Care China.

[12] Du SH (2016). Exploration of changes of mandible and maxilla and soft tissues of patients with class II malocclusion after correction based on double jaw pillow and muscular activator. J Clin Med Literat (Electr Edit).

[13] Zhuang Y, Yin TG (2015). Comparative studies of Mini-twin block appliance and muscular activator in early treatment of class II division 1 malocclusion. Chin Mod Doct.

[14] Zhang HJ, Sun XJ, Zhang H, Xie H (2015). Correction of skeletal class III malocclusion with maxillary protraction. Chin J Aesthet Plast Surg.

[15] He YQ, Chen H (2015). Application of rapid prototyping with 3D printing technology in plastic repair of cranial and maxillofacial deformity. Chin J Aesthet Med.

[16] Schubert C, Langeveld MC, Donoso LA (2014). Innovations in 3D printing:a 3D overview from optics to organs. Br J Ophthalmol.

[17] Zhou LB, Shang HT, He LS, Bo B, Liu GC, Liu YP (2010). Accurate reconstruction of discontinuous mandible using a reverse engineering/computer-aided design/rapid prototyping technique:a preliminary clinical study. J Oral Maxillofac Surg.

[18] Shen ML, Wang XD (2016). Application of digital three-dimensional surgical simulation system in specialist training of orthognathic surgery. Chin Med Educ Tech.

[19] Hu M, Tan XY, Yan RZ, Liu CK, Li XF, Liu C (2014). Progress of application of 3D printing technology in the oral and maxillofacial surgery. Chin J Pract Stomatol.

[20] Zhang H, An YJ, Peng WL (2016). Analysis of correction effect of tooth extraction on the width of dental arch of patients with class II division I malocclusion. Contemp Med.

[21] Wang YC (2014). Analysis of recovery of dental arch width and morphology of class I malocclusion after tooth extraction. Chin J Mod Drug Appl.

[22] El Hajj N, Bassil-Nassif N, Tauk A, Mouhanna-Fattal C, Bouserhal JP (2017). Maxillary and mandibular contribution to the establishment of class II malocclusion in an adult Lebanese population. Int Orthod.

[23] Wang Y, Qin P, Du YH (2011). A longitudinal study of mandible growth of Angle Class II division 2 malocclusion from mixed dentition to permanent dentition. W Chin J Stomatol.

[24] Frye L, Diedrich PR, Kinzinger GS (2009). Class II treatment with fixed functional orthodontic appliances before and after the pubertal growth peak - a cephalometric study to evaluate differential therapeutic effects. J Orofac Orthop.

[25] Tian NX, Huang JF (1988). Growth changes of craniofacial morphology of anterior teeth with malocclusion with deep overburden jaws. Chin J Stomatol.

